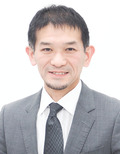# Best Reviewers Award for 2021

**DOI:** 10.1002/deo2.116

**Published:** 2022-04-05

The DEN Open Best Reviewers Award is an annual prize which recognizes the very best reviewers for their high‐quality reviews and dedication. Over 170 scholars served as reviewers in 2021, and we are pleased to announce 20 winners who have been selected based on the following criteria:

1. Invitation acceptance rate: 80% and over.
2. Number of completed reviews: 1.5 or above; How to calculate the number of the articles reviewed:
  - Reviews/Original Articles ——— x1
  - Case Reports ——— x0.5
3. Top 20 reviewers whose average scores for review: eight points maximum and two points minimum, Quality (5‐point scale) + Timeliness (3‐point scale).

Review period: November 2020 to December 2021

**Table:** 

**Hideyuki Chiba** Department of Gastroenterology, Omori Red Cross Hospital, Tokyo, Japan 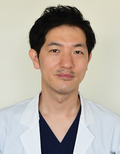	**Mitsuru Esaki** Department of Medicine and Bioregulatory Science, Graduate School of Medical Sciences, Kyushu University, Fukuoka, Japan 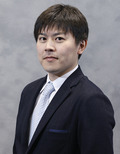	**Toshio Fujisawa** Department of Gastroenterology, Graduate School of Medicine, Juntendo University, Tokyo, Japan 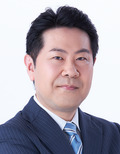	**Kazuo Hara** Department of Gastroenterology, Aichi Cancer Center, Aichi, Japan 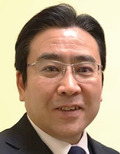
**Takuto Hikichi** Department of Endoscopy, Fukushima Medical University Hospital, Fukushima, Japan 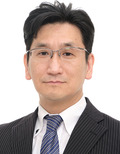	**Yusuke Horiuchi** Deparment of Gastroenterology, Cancer Institute Hospital of Japanese Foundation for Cancer Research, Tokyo, Japan 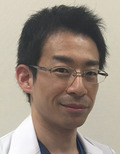	**Tadahisa Inoue** Department of Gastroenterology, Aichi Medical University, Aichi, Japan 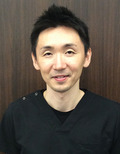	**Takuji Iwashita** First Department of Internal Medicine, Gifu University Hospital, Gifu, Japan 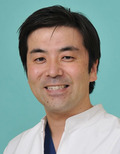

**Table:** 

**Yoshihide Kanno** Department of Gastroenterology, Sendai City Medical Center, Miyagi, Japan 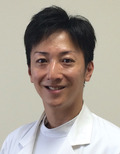	**Motohiko Kato** Division of Research and Development for Minimally Invasive Treatment, Cancer Center, Keio University School of Medicine, Tokyo, Japan 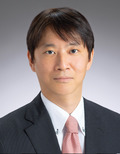	**Yoshiyasu Kono** Department of Gastroenterology and Hepatology, Faculty of Medicine, Dentistry and Pharmaceutical Sciences, Okayama University, Okayama, Japan 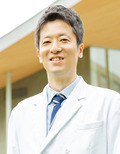	**Mai Ego Makiguchi** Endoscopy Division, National Cancer Center Hospital, Tokyo, Japan 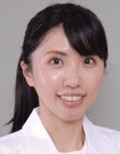
**Kosuke Minaga** Department of Gastroenterology and Hepatology, Kindai University Faculty of Medicine, Osaka, Japan 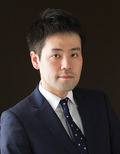	**Kazunari Nakahara** Department of Gastroenterology and Hepatology, St. Marianna University School of Medicine, Kanagawa, Japan 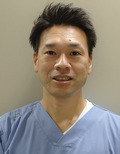	**Yousuke Nakai** Department of Gastroenterology, Graduate School of Medicine, The University of Tokyo, Tokyo, Japan 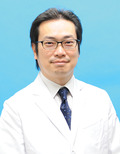	**Hiroki Sato** Division of Gastroenterology & Hepatology, Mayo Clinic, Minnesota, USA 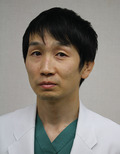
**Sho Suzuki** Department of Gastroenterology, International University of Health and Welfare Ichikawa Hospital, Chiba, Japan 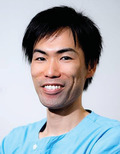	**Yosuke Toya** Division of Gastroenterology, Department of Internal Medicine, Iwate Medical University, Iwate, Japan 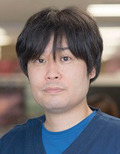	**Shunichi Yanai** Division of Gastroenterology, Department of Internal Medicine, Iwate Medical University, Iwate, Japan 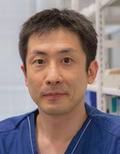	**Naohisa Yoshida** Department of Molecular Gastroenterology and Hepatology, Kyoto Prefectural University of Medicine, Kyoto, Japan